# P-1734. Epidemiologic Characterization of Histoplasmosis in Costa Rica: A 16-Year Nationwide Study

**DOI:** 10.1093/ofid/ofaf695.1905

**Published:** 2026-01-11

**Authors:** Jose A Castro Cordero, Juan Villalobos Vindas, Alvaro A Aviles Montoya, Carlos Ramírez Valverde, Saúl Quirós Cárdenas, Laura Villalobos González

**Affiliations:** Caja Costarricense de Seguro Social, Uruca, San Jose, Costa Rica; Caja Costarricense de Seguro Social, Uruca, San Jose, Costa Rica; Caja Costarricense de Seguro Social, Uruca, San Jose, Costa Rica; Caja Costarricense del Seguro Social, San José, San Jose, Costa Rica; CCSS, San Jose, San Jose, Costa Rica; Caja Costarricense de Seguro Social, Uruca, San Jose, Costa Rica

## Abstract

**Background:**

Histoplasmosis remains an important endemic mycosis in tropical countries, yet comprehensive epidemiological data from Central America are limited. We aimed to describe the epidemiologic characteristics of histoplasmosis in Costa Rica during a 16-year period.

**Figure:**
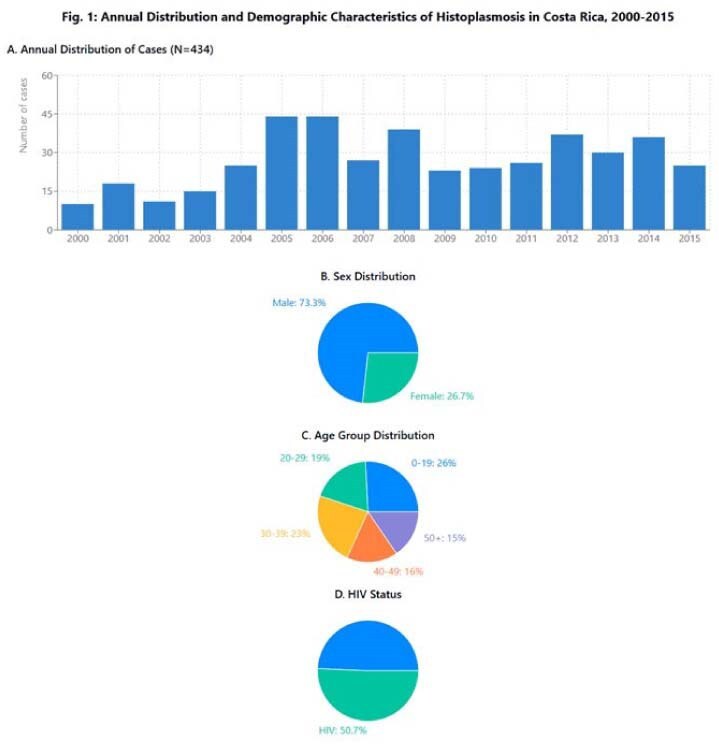
Annual Distribution and Demographic Characteristics Annual Distribution and Demographic Characteristics of Histoplasmosis in Costa Rica, 2000-2015

**Figure:**
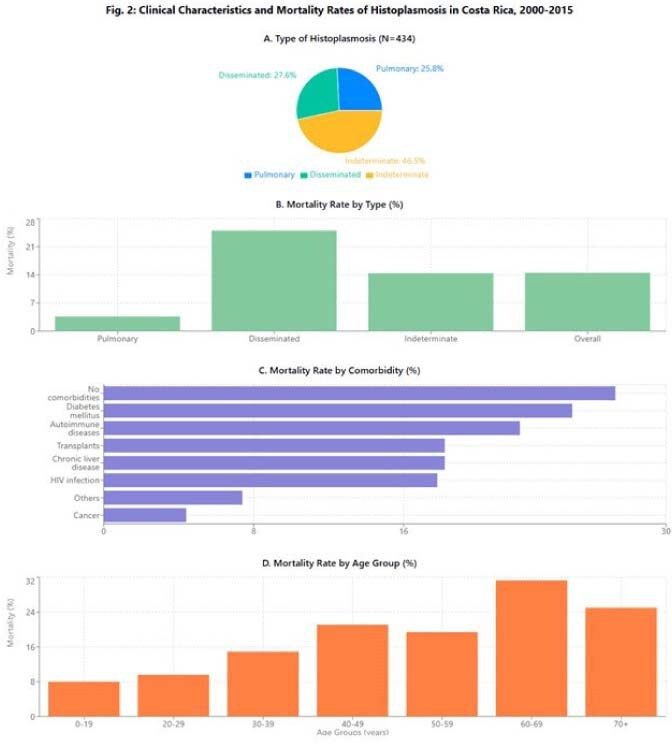
Clinical Characteristics and Mortality Rates Clinical Characteristics and Mortality Rates of Histoplasmosis in Costa Rica, 2000-2015

**Methods:**

We conducted a retrospective, descriptive study analyzing all histoplasmosis cases (n=434) registered in the national hospital discharge database of Costa Rica's Social Security System from 2000-2015. We calculated cumulative incidence per 100,000 population, analyzed demographic distribution, comorbidities, clinical presentations, and in-hospital mortality.

**Figure:**
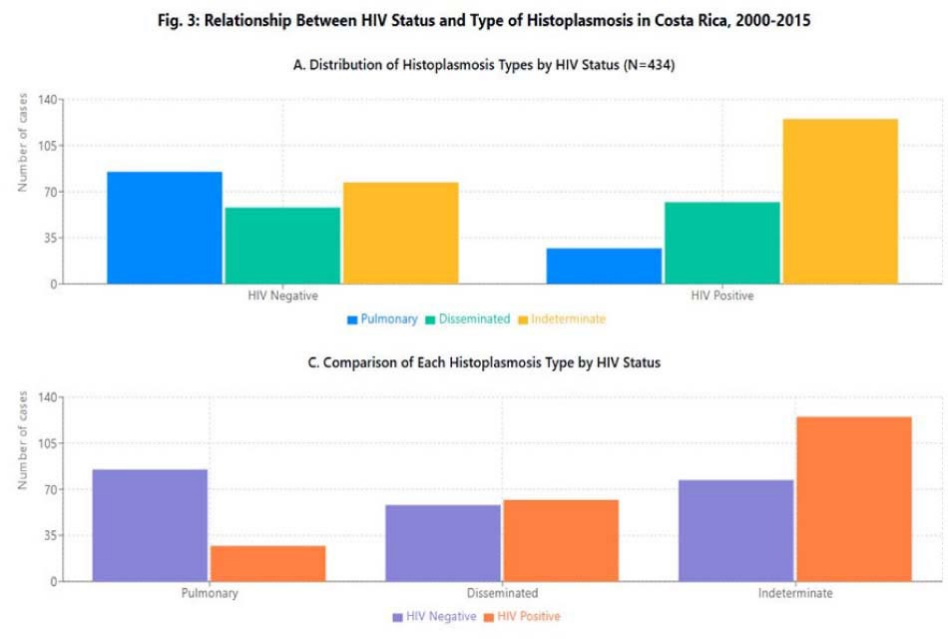
Relationship Between HIV Status and Type of Histoplasmosis Relationship Between HIV Status and Type of Histoplasmosis in Costa Rica, 2000-2015

**Figure:**
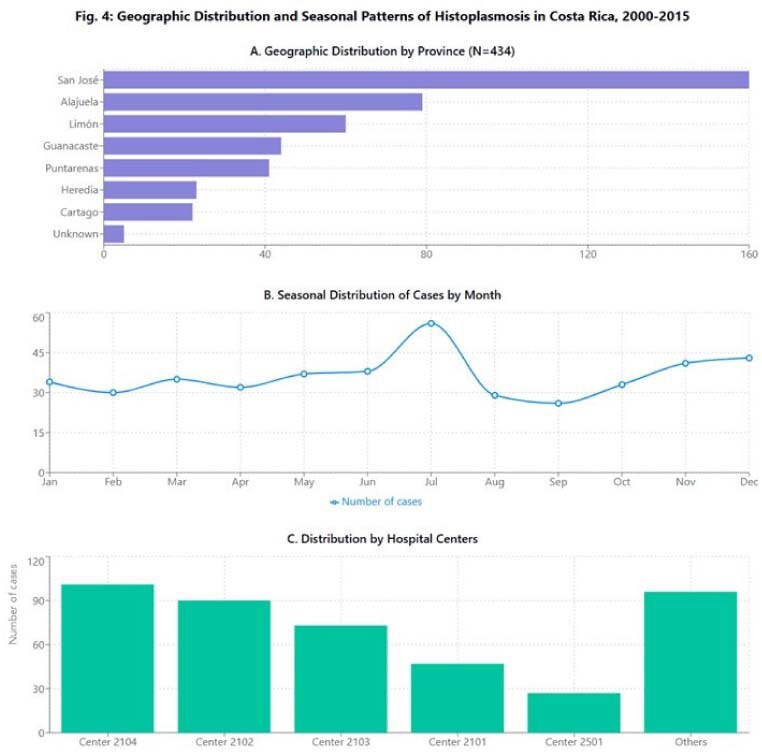
Geographic Distribution and Seasonal Patterns of Histoplasmosis Geographic Distribution and Seasonal Patterns of Histoplasmosis in Costa Rica, 2000-2015

**Results:**

The cumulative incidence was 0.62 cases/100,000 person-years, with temporal variations showing an initial increase followed by a slight decline. Males were predominantly affected (73.3%; RR=2.68, 95%CI:2.17-3.31). Median age was 31 years (IQR:18-43), with 26% of cases in patients < 19 years and 59% between 20-49 years. HIV infection was present in 49.3% of patients, while 46% had other comorbidities including cancer (5.3%), chronic liver disease (2.5%), and kidney transplant (2.5%); 5.1% had no underlying conditions. Clinical presentations were classified as pulmonary (25.8%), disseminated (27.6%), and undetermined (46.5%). Overall in-hospital mortality was 14.5%, being significantly higher in disseminated disease (25%) and elderly patients (31.3% in 60-69 age group). Surprisingly, patients without documented comorbidities had the highest mortality rate (27.3%). Geographic distribution showed predominance in San José (36.9%), Alajuela (18.2%), and Limón (13.8%) provinces. Incidence peaks occurred during 2005-2006 (44 cases/year), with July showing the highest diagnostic frequency (12.9%).

**Conclusion:**

Histoplasmosis in Costa Rica maintains a relatively stable incidence, primarily affecting young adults with a strong association with HIV infection. The unexpectedly high mortality in patients without apparent risk factors suggests potential delayed diagnosis or unidentified factors requiring further investigation. The disseminated form carries the highest mortality risk, emphasizing the need for early detection strategies, particularly in high-risk populations.

**Disclosures:**

All Authors: No reported disclosures

